# The composition and mode of delivery of diabetes‐related footcare education provided by podiatrists in Australia and Aotearoa (New Zealand): A systematic review

**DOI:** 10.1002/jfa2.70009

**Published:** 2024-11-11

**Authors:** Maasooma Al Husaini, Angela Searle, Vivienne Chuter

**Affiliations:** ^1^ School of Health Sciences Western Sydney University Campbelltown Sydney Australia

**Keywords:** amputation, diabetes, education, foot care, foot ulcer, podiatry

## Abstract

**Introduction:**

Diabetes‐related foot disease (DFD) is a significant and costly complication of diabetes in Australia and Aotearoa New Zealand (NZ). Diabetes footcare education is considered a cornerstone of DFD prevention and management, with podiatrists playing a key role in education provision. This systematic review evaluated the nature and composition of diabetes footcare education provided by podiatrists to people living with diabetes in Australia and NZ.

**Methods:**

Medline, EBSCO, Megafile Ultimate and Cochrane library databases were conducted from inception until January 31, 2024 to identify studies reporting on the mode of delivery and composition, including frequency, of diabetes footcare education provided to people with diabetes by podiatrists in Australia and NZ.

**Results:**

From a total of 226 abstracts screened, 4 studies with 878 participants were included. Three studies were from Australia and 1 from NZ. Studies included podiatrists in both private and public health sectors and used cross‐sectional web‐based surveys or observation. Components of diabetes footcare education included education on neuropathy and vascular foot health, footwear and general foot health/hygiene. This education was provided by podiatrists from both countries routinely. Verbal education was the most frequently used method of delivery. There was no significant difference between content, mode of delivery and frequency of diabetes footcare education between private and public practitioners in either country. No studies reported on culturally responsive content or education delivery methods.

**Conclusion:**

There are little available data on the composition or mode of delivery of diabetes footcare education provided by podiatrists in Australia and NZ to people living with diabetes. A range of footcare education is provided, most frequently verbally. Further qualitative research is required to conclusively establish the composition and delivery methods used for diabetes footcare education provided by podiatrists. In addition, the provision of culturally responsive diabetes footcare education and availability of related culturally responsive supporting resources is yet to be established.

## 1 INTRODUCTION

It is estimated that there are 537 million people living with diabetes worldwide, with this expected to increase to 783 million people by 2045 [1]. Diabetes‐related foot disease (DFD) is a significant and costly complication of diabetes encompassing a range of conditions that affect the lower limb including peripheral neuropathy, peripheral artery disease, infection, gangrene, diabetes‐related foot ulcer (DFU) and amputation [2]. One of the most recognised DFD complications, DFU affects up to 34% of people with diabetes in their lifetime and frequently precedes minor and major amputation [3]. Five year mortality rates for people with DFU are over 30% and over 50% for those undergoing subsequent major amputation [4].

In Australia in 2021, 1 in 20 people and almost 8% of Aboriginal and Torres Strait Islander Peoples were living with diabetes [5]. DFD is estimated to affect 50,000 Australians annually, at a cost of over $1.6 billion [6, 7, 8]. Similarly, in Aotearoa, New Zealand (NZ) diabetes is estimated to affect 228,000 individuals with a total cost of $NZD2.1 billion per annum [9]. Furthermore, in both countries, the profound and ongoing impacts of colonisation on the health and well‐being of Indigenous Peoples are reflected by disproportionately high rates of diabetes and diabetes complications including DFD [9, 10]. Aboriginal and Torres Strait Islander Peoples of Australia and Māori Peoples of NZ are more likely to develop diabetes at a younger age and to experience more severe complications from the disease including higher rates of DFU and amputation than their non‐Indigenous counterparts, and to have lower participation in diabetes footcare services and inadequate access to culturally safe care [9, 10, 11, 12, 13, 14].

Current guidelines from the International Working Group on the Diabetic Foot (IWGDF) recommend provision of education as a cornerstone of DFD prevention [15, 16]. They suggest well‐structured footcare education aimed at improving self‐care abilities and self‐protective behaviors of people living with diabetes that is delivered in a motivating and organised manner [15, 16]. Such education is widely considered to be instrumental in reducing rates of DFD and includes educating people living with diabetes about general footcare, managing risk associated with changes in sensation caused by neuropathy, wound care and use of properly fitting protective footwear.

Supporting these guideline recommendations, systematic review of the effect of education interventions on DFD outcomes has shown a reduction in DFD with use of intensive education strategies [17]. However, research suggests that methods of education delivery by podiatrists are inconsistent, education covers a broad range of topics and there is a lack of evaluation of effectiveness [18]. For example, a survey of a small proportion of Australian registered podiatrists (10%) on the content and the methods of delivery of the diabetes footcare education they provided reported variable approaches are used [18]. These included verbal, written, visual formats and use of interactive programs as well as combinations of these methods, with a wide range of topics relating to diabetes care included [18]. Having a comprehensive understanding of the current practices in provision of diabetes footcare education by podiatrists to people living with diabetes is essential to identify areas to improve practice and to underpin further assessment of comparative effectiveness of diabetes footcare education content and methods of education delivery. Therefore, the aim of this review was to systematically examine the literature reporting on the nature and composition of diabetes footcare education provided by podiatrists to people living with diabetes in Australia and NZ.

## 2 MATERIALS AND METHODS

This review was conducted in accordance with the Preferred Reporting Items for Systematic Review and Meta‐Analysis (PRISMA) (PROSPERO ID: CRD42024512700).

### 2.1 Search strategy

Title and abstract searches of Medline, Embase and CINAHL databases were conducted from inception until January 31, 2024 to identify studies reporting on the nature and composition of diabetes footcare education provided to people with diabetes by podiatrists in Australia and NZ. Search strings are provided in Supplementary file 1.

### 2.2 Eligibility assessment

Eligible studies had to be published in peer reviewed journals and report on the mode of delivery and composition of diabetes footcare education provided to people living with diabetes by podiatrists in the private or the public health sectors in Australia or NZ. Studies could be quantitative, qualitative or mixed methods.

There was no restriction on age of participants, years of experience of the podiatrist, year of publication or language. Studies were excluded if they included other health practitioners and data for podiatrists were not reported separately or could not be retrieved from the authors. In addition, studies including global data where data for Australia and NZ were not reported separately or could not be retrieved, and studies on diabetes education that did not include footcare were excluded.

### 2.3 Quality assessment

As this review included both qualitative and quantitative studies, the Mixed Methods Appraisal Tool (MMAT) 2018 [19] was used. The MMAT assesses the quality of articles against a number of key methodological criteria including clarity of the question, applicability of data to answer the research question, appropriate study design, inclusion of relevant studies, rigor of studies, clarity and precision of results, consideration of important outcomes, confounding factors, and bias minimisation. Two authors (MA and VC) independently appraised the articles, and any discrepancies were discussed until a consensus was reached. Where there was disagreement, arbitration was to be performed by a third reviewer (AS) however this was not required.

### 2.4 Data extraction

Titles and abstracts were screened independently by two authors (MA and VC). Full‐text articles of included abstracts were retrieved and assessed for inclusion independently by the same two reviewers with any conflicts to be resolved by a third author (AS) however this was not required. Hand searching of the reference list of included articles was also undertaken. Data extraction was performed by one author (MA) using a customized data extraction form and cross‐checked by another author (VC).

### 2.5 Data analysis

Qualitative data were to be assessed via a narrative synthesis with descriptive statistics and frequency analysis used to summarise the quantitative outcomes relating to mode or composition of delivery of diabetes footcare education where possible. It was pre‐determined that a meta‐analysis of quantitative outcomes relating to mode or composition of delivery by country and by podiatrist characteristics for example, private or public sector employment, would be undertaken where there were available data. If a meta‐analysis was not possible, it was our intention to conduct a narrative analysis of available data.

## 3 RESULTS

**FIGURE 1:**
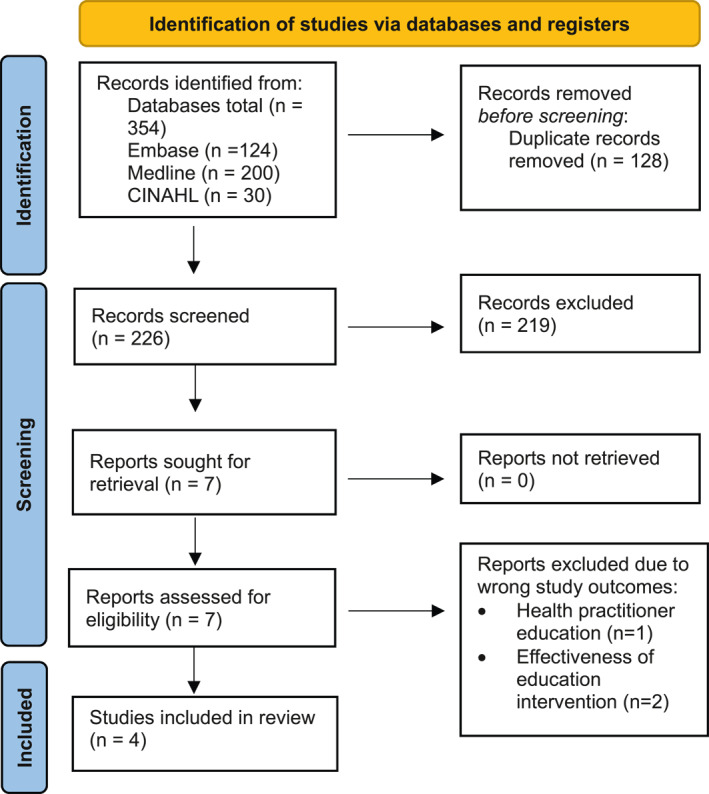
Preferred reporting items for systematic reviews and meta‐analyses flow diagram.

A total of 354 titles and abstracts were identified from the empirical search. After 128 duplicates were removed there were 226 publications remaining for eligibility screening and 219 were excluded. Of the 7 full text publications that were retrieved 3 were excluded due to having wrong study outcomes [20, 21, 22], leaving 4 publications to be included in this review (Figure 1).

### 3.1 Study characteristics

The 4 included studies had a total of 878 participants (Table 1). Studies included podiatrists in both private and public health sectors and used cross‐sectional web‐based surveys or observation [18, 23, 27, 28]. Three studies were from Australia and 1 was from NZ. Participant recruitment for 3 of the studies was done via advertising through professional organisations, social media and professional events (e.g., conferences, specialist group meetings) and 1 study recruited podiatrists in a single public podiatry department. Two studies reported on diabetes footcare education as a component of a survey on diabetes foot management [23, 27], 1 study included a survey that only investigated diabetes footcare education [18] and the final study investigated diabetes footcare education as part of a scheduled podiatry appointment [28]. One study developed a customised 36‐item Australian Diabetic Foot Management survey employing seven‐point Likert scales (0 = Never; 7 = Always) to measure multiple aspects of best practice diabetes foot management [27]. Similarly Jepson et al. [23], used a 37‐item web‐based survey with a 5‐point Likert scale (0 = always; 5 = never) based on the IWGDF 2019 prevention guidelines [29] to assess multiple areas of practice. In 1 study Yuncken et al. [18], used a customised web‐based survey to collect information on how podiatrists provide diabetes footcare education within the clinical setting. The remaining study used observation and verbal questioning to determine education content and delivery and participant‐podiatrist concordance with the key message from the consultation [28].

**TABLE 1:** Characteristics and outcomes of the included studies.

Citation	Study description	Study participants	Study objectives	Average number of people with DFU treated/week	Diabetes footcare education composition & frequency	Mode of delivery
Jepson (2023) [23]	Cross‐sectional observational web‐based survey distributed through professional podiatry networks to NZ podiatrists between Nov & Dec 2022.	*N* = 77 responses, 52 analyzed. Podiatrists of different ethnicities working across different regions of NZ. Podiatry practice: Private: 38 High risk foot service: 11 Research: 1 Education: 1 Other: 1	To identify alignment between assessment and management strategies used by NZ podiatrists in the prevention of DFD, and the international guideline recommendations.	Private: 1–5 Public: 21–30	Median Likert agreement value with interquartile range (IQR) for 5‐point Likert scale responses: Always (1), Often (2), Sometimes (3), Seldom (4), Never (5) Foot care Medium risk Total: 1 (1−1) Private: 1 (1−1) Public: 1 (1−1), *p* = 0.84 High risk Total: 1 (1−1) Private: 1 (1−1) Public: 1 (1−1), *p* = 0.84 Foot hygiene Medium risk Total: 1 (1−2) Private: 1 (1−2) Public: 1 (1−2), *p* = 0.30 High risk Total: 1 (1−2) Private: 1 (1−2) Public: 1 (1−1), *p* = 0.53 Footwear Medium risk Total: 1 (1−2) Private: 1 (1−2) Public: 1 (1−2), *p* = 0.52 High risk Total: 1 (1−1) Private: 1 (1−2) Public: 1 (1−1), *p* = 0.60 First aid Medium risk Total: 1 (1−3) Private: 2 (1−2) Public: 1 (1−3), *p* = 0.18 High risk Total: 1 (1−2) Private: 2 (1−3) Public: 1 (1−1), *p* = 0.21	One‐on‐one verbal education n (%) Effect of diabetes on the lower limb: Private: 40 (98) Public: 11 (100), *p* = 0.99 Preventative foot care: Private: 41 (100) Public: 10 (91), *p* = 0.70 Management of special problems: Private: 37 (90) Public: 10 (91), *p* = 0.90 Resources & handouts n (%): Effect of diabetes on the lower limb: Private: 16 (38) Public: 5 (45), *p* = 0.67 Preventative foot care: Private: 15 (24) Public: 4 (25), *p* = 0.89 Management of special problems: Private: 17 (41) Public: 5 (12), *p* = 0.78 Links to external network n (%): Effect of diabetes on the lower limb: Private: 7 (17) Public: 3 (26), *p* = 0.49 Preventative foot care: Private: 7 (17) Public: 2 (18), *p* = 0.79 Management of special problems: Private: 5 (12) Public: 1 (9), *p* = 0.97 Structured education n (%): Effect of diabetes on the lower limb: Private: 4 (10) Public: 0 (0), *p* = 0.19 Preventative foot care: Private: 2 (5) Public: 0 (0), *p* = 0.26 Management of special problems: Private: 3 (7) Public: 1 (9), *p* = 0.75
Quinton (2015) [27]	Cross‐sectional study of Australian diabetic foot management surveys distributed to Australian podiatrists between May and June 2013	*N* = 311 Male: 89 Female: 222 Podiatry practice: Metropolitan: 188 Regional and rural: 123 Public: 158 Private: 153	To examine Australian podiatrists' diabetes related foot management compared with best practice recommendations by the Australian National Health Medical Research Council	Private: 1–5 Public: 6–10	Likert scale (median and interquartile range): 1 = never (0%), 2 = very rarely (1%–20%), 3 = rarely (21%–40%), 4 = sometimes (41%–60%), 5 = often (61%–80%), 6 = very often (81%–99%), 7 = always (100%) Provide foot care education to prevent foot complications: Private: 7 (6–7), Public: 7 (6–7), *p* = 0.709 Provide or recommend footwear to prevent foot complications: Private: 6 (6–7), Public: 6 (6–7), *p* = 0.927	Not reported
Yuncken (2020) [18]	Cross‐sectional cohort study via online survey of Australian podiatrists from April to August 2018	*N* = 512 Female: 329 Male: 181 Prefer not to answer: 2 Acute setting: 83 Sub‐acute setting: 24 Community health: 104 Private: 280 Other: 21	To describe the content, mode & methods of foot related diabetes education delivered by Australian podiatrists to people with diabetes	Not reported	Education topics in unprompted versus prompted questions % Vascular Unprompted: 50 Prompted: 96, *p* = 0.99 Neuropathy Unprompted: 52 Prompted: 98, *p* = 0.75 Ulceration risks Unprompted: 23 Prompted: 93, *p*=<0.01 Footwear Unprompted: 23 Prompted: 96, *p*=<0.01 General skin & nail care Unprompted: 46 Prompted: 90, *p* < 0.01 Blood sugar levels Unprompted: 2 Prompted: 87, *p* < 0.01 Physical activity Unprompted: <1 Prompted: 73, *p* < 0.01 Smoking Unprompted: <1 Prompted: 65, *p*=<0.01 Dietary Unprompted: <1 Prompted: 56, *p*=<0.01 Medication Unprompted: <1 Prompted: 0, *p* = 0.32 Referrals Unprompted: <1 Prompted: 0, *p* = 0.02	Delivery mode unprompted versus prompted questions % Written Unprompted: 0 Prompted: 47, *p* < 0.01 Verbal Unprompted: 46 Prompted: 94, *p* < 0.01 Visual aid/video Unprompted: 0 Prompted: 14, *p* < 0.01 Handout individual Unprompted: 0 Prompted: <1, *p* < 0.01 Handout proforma Unprompted: 0 Prompted: 51, *p* < 0.01 Verbal & written Unprompted: 44 Prompted: 0, *p* < 0.01 Verbal & written & visual Unprompted: <1 Prompted: 0, *p* < 0.01 Verbal & visual Unprompted: <1 Prompted: 0, *p* < 0.01 No education Unprompted: <1 Prompted: 0, *p* = 0.33
Yuncken et al. (2018) [28]	Prospective cohort study of podiatrists & their patients in a public health clinic	*N* = 3 podiatrists, 24 patients	To determine podiatrist education methods & content & patient recall of footcare education over 6 months	Not reported	Topics (number of times covered) Vascular: 14 Footwear: 14 Wound care: 16 Neuropathy: 17 General foot care: 14 Follow up care: 17 Topics/consultation (*n*) 4 topics = 10 3 topics = 6 5 topics = 5	Verbal: 100% Written individual: 9% Written proforma: 0% Difference in key education message patient versus podiatrist at post appointment: 58% 6 months: 63%

Abbreviations: %, percentage; DFD, Diabetes‐related foot disease; IQR, interquartile range; *n*, number; NZ, Aotearoa New Zealand.

### 3.2 Quality appraisal

Included studies met the majority of methodological quality criteria included in the MMAT (Table 2). One study reported inconsistencies between quantitative and qualitative survey results [18]. These differences were assessed for statistical significance with some discussion of the reasons for the divergent results included, however, the reasoning behind the design of the survey lacked clarity. One study documented the diabetes footcare education methods of 3 podiatrists in a single public health clinic with an observer in the consultation room, and these factors could result in the findings not being reflective of the practices of podiatrists generally [28].

**TABLE 2:** Mixed methods appraisal tool (MMAT), version 2018.

	Jepson (2023)			Quinton (2015)			Yuncken (2020)			Yuncken (2018)		
Methodological quality criteria	Yes	No	Can't tell	Yes	No	Can't tell	Yes	No	Can't tell	Yes	No	Can't tell
S1. Are there clear research questions?	X			X			X			X		
S2. Do the collected data allow to address the research questions?	X			X			X			X		
5.1. Is there an adequate rationale for using a mixed methods design to address the research question?	X			*X*			*X*			X		
5.2. Are the different components of the study effectively integrated to answer the research question?	X			*X*			*X*			X		
5.3. Are the outputs of the integration of qualitative and quantitative components adequately interpreted?	X			*X*			*X*			X		
5.4. Are divergences and inconsistencies between quantitative and qualitative results adequately addressed?	X			*X*			*X*			X		
5.5. Do the different components of the study adhere to the quality criteria of each tradition of the methods involved?	X			*X*			*X*			X		

### 3.3 Composition of education

One study reported the composition of diabetes footcare information provided in NZ by topic and DFU risk status (Table 1) [23]. Topics of foot care, foot hygiene and footwear were reported to be ‘always' provided by practitioners in private and public sectors. First aid (undefined by the study authors but related to DFD prevention education) was reported to be often delivered by private practitioners and always delivered by public practitioners. There was no significant difference between private and public sector practitioners for frequency of provision of any topic.

Three studies reported on composition and frequency of diabetes footcare education provided by podiatrists in Australia (Table 1) [18, 27, 28]. Quinton et al. [27], reported on how often podiatrists provide footcare education using a 7 item Likert scale. Public and private sector median responses showed no difference between the groups, with scores of 7 (always [100%]) for providing education to prevent foot complications, and 6 (very often [81%–99%]) for providing footwear recommendations to prevent foot complications. Yuncken et al. [18], used prompts (where responses are selected from a list provided in the survey) and open‐ended survey questions to report on footcare education topics provided to people living with diabetes. Results between prompted responses and open‐ended questions were discordant, with lower figures reported in the open‐ended questions. Prompted responses demonstrated that education on neuropathy and vascular foot health, footwear and general foot health and foot ulcer risk were provided routinely by 90% or more of respondents. However, open‐ended responses revealed education on neuropathy, vascular foot health and general foot health education was provided by approximately 50% of respondents, and footwear education and foot ulcer risk education by only 23% of respondents. The authors noted that participant responses indicated some confusion regarding an overlap between prompted and open‐ended questions and suggested this may have biased outcomes. Discussion of 2 or more education topics per consultation were reported by 89% of practitioners. A further pilot study by Yuncken et al. [28], of 3 podiatrists, found that education regarding wound care, neuropathy and follow up care were the most common footcare education topics covered during the consultation. In the majority of consultations between 3 and 5 education topics were discussed.

### 3.4 Mode of education delivery

Three studies reported on mode of delivery of footcare education (Table 1) [18, 23, 28]. Jepson et al. [23], reported on NZ practitioners delivery modes relating to 3 education topics (1) the nature and effect of education on the lower limb, (2) preventative foot care behaviors including hygiene and foot inspection, and (3) management of special problems (particular to the patient). Verbal education was most frequently provided across all 3 education domains with 90%–100% of practitioners using this technique. Structured education was used the least with the percentage of practitioners using this across the three domains ranging from 0% to 10%. No differences were observed in composition of education between public and private sector podiatrists for any education domain. Yuncken et al. [18], investigated modes of delivery of diabetes footcare education delivered by podiatrists in Australia using prompted and unprompted questions which again displayed differing results. Prompted responses indicated that verbal education was used by 94% of respondents compared to a result of 46% using open‐ended questions. Nevertheless, similar to the NZ data, verbal education was the most frequently used form of education delivery. Use of written and handout/proforma were the next most used mode of education at 47% and 51% respectively. In the final study the 3 podiatrists all provided verbal education, with only 2 study participants receiving written individual education [28].

## 4 DISCUSSION

This systematic review investigated the composition and mode of delivery of diabetes‐related footcare education provided by podiatrists in Australia and NZ to prevent DFD. These data demonstrated that podiatrists cover a broad spectrum of content related to diabetes‐related footcare, with data from both countries identifying verbal education is the most frequently utilised method of education delivery [18, 23, 28]. However, the nature of the verbal education provided by podiatrists could not be clearly established from the available data and is likely to influence effectiveness of the education intervention. For example, techniques such as motivational interviewing have been linked to more effective education that supports behavioral change in addition to improving knowledge of diabetes foot care [26]. Recent research has also shown a collaborative patient‐centered approach to diabetes foot and wound care education resulted in greater knowledge retention and increased self‐care behaviors in people living with diabetes compared to didactically delivered education [30].

Most significantly, this systematic review has identified there are very little data investigating diabetes footcare education provision in Australia and NZ [18, 23, 27, 28]. In addition, interpretation of available data is largely restricted to survey data with small sample sizes relative to the size of the profession in each country. Furthermore, the discordant responses between prompted and unprompted response survey questions reported by Yuncken et al. [18], make interpretation of current practice in Australia more difficult. In addition, use of Likert scales by included studies [23, 27] to measure frequency of diabetes footcare education does not explain factors that may influence the frequency. To date there has been no published in‐depth qualitative evaluation of diabetes footcare education practices by podiatrists in either country, and therefore the alignment of current practices with diabetes footcare guidelines is difficult to determine.

Diabetes footcare education is considered to be effective at reducing DFD however the extent to which this is the case is yet to be conclusively established and creates further challenges in ensuring effective education content and delivery methods [31]. Recent meta‐analysis of randomized controlled trials investigating the effectiveness of education interventions for DFD prevention demonstrated risk of a foot ulcer was halved however with notable imprecision of the estimate of effect (OR 0.54; 95% CI 0.29–1.00) and moderate study heterogeneity [31]. Risk of any amputation was also reduced (OR 0.34; 95% CI 0.13, 0.88, *I* ^2^ = 38%) and DFD knowledge (13 of 16 trials) and self‐care behavior scores (19 of 20 trials) improved. However, 28 of 29 studies had moderate to high risk of bias with the authors noting the need for long‐term high‐quality trials [31]. Similarly systematic review of the effectiveness of footcare education on self‐care and self‐efficacy outcomes for people living with diabetes concluded that although available RCTs were heterogeneous and of low to moderate quality, the majority of included studies reported improvement in foot self‐care and self‐efficacy scores [32].

Previous reviews of the effectiveness of patient education on preventing DFU and amputation have also had mixed findings. A 2014 Cochrane review concluded there were little available evidence to support the effectiveness of education on prevention of DFU with low quality studies assessing education interventions likely to be a key factor in inconsistent findings [33]. Of note the review found one RCT of adequate methodological quality that showed limited patient education did not result in any beneficial effect on prevention of DFU. Similarly, a 2018 systematic review comparing intensive and brief patient education strategies in preventing and reducing the incidence or recurrence of DFU found a statistically reduced risk of incidence of DFU in those receiving intensive education, however heterogeneity of included studies was high [17]. These findings suggest that further investigation of more comprehensive/intensive preventative education interventions needs to be undertaken to determine their comparative effectiveness.

Current IWGDF Guidelines for provision of education for prevention of DFD emphasize the importance of providing structured education that is presented in an organized manner and is repeated to people living with diabetes, their family and health carers [15, 16]. Further recommendations relate to providing culturally appropriate health care education that is person centered. Although not the primary aim of this review, it was surprising that none of the included studies reported on provision of culturally responsive diabetes footcare education. In both countries, where rates of DFU and amputation are disproportionately high for Aboriginal and Torres Strait Islander and Māori Peoples, understanding the practices of podiatrists in provision of culturally responsive diabetes footcare education and establishing availability of culturally responsive diabetes footcare education resources is essential. Previous research has established that culturally responsive healthcare education is effective in supporting better health outcomes [34]. Meta‐analysis has demonstrated significant sustained (over 24 months) improvements in participant knowledge of diabetes and glycaemic control (HbA1c) after the delivery of culturally responsive health education in comparison to “conventional” care [34]. Similarly, for Aboriginal and Torres Strait Islander Peoples, self‐reported diabetes footcare knowledge has been shown to improve following engagement with culturally safe, Community‐led DFD prevention services [35]. These findings support the need for Community‐led interventions that appropriately reflect the heterogeneity of Indigenous populations and rebut non‐inclusive monocultural health care approaches and evaluation of those interventions [36]. Historical research has identified that diabetes footcare education booklets and posters developed specifically for First Nations Peoples in Australia were well received by Aboriginal people from a range of Communities [37]. However, the effectiveness of such resources in prevention of DFU is unclear and there is a remaining need to develop Community‐based resources.

Although this present review focused on footcare education, it found that Australian podiatrists reported a wide range of education content was delivered to people living with diabetes [18]. This education included both diabetes footcare education and general diabetes education including diabetes management, physical activity education and smoking cessation education. Similarly, the studies included in this review reported a range of modes of education were used by podiatrists with the most consistently reported being verbal education and few relying on structured education sessions [18, 23]. These findings are consistent with a recent systematic review of the efficacy of diabetes education for people with Type 2 diabetes which reported a range of education techniques being used in primary health care including group education, individual education and mixed education strategies. Education sessions were provided by a range of health professionals and were of differing durations, ranging from 1 day to appointments scheduled over 24 months [25]. The content of the education programs was also varied, including mechanisms of diabetes, signs and symptoms, acute and chronic complications and risk factor reduction. Despite the variability between studies, the review showed most education interventions were effective on at least some outcome variables (e.g., metabolic outcomes) however it is unknown whether long term changes can be achieved [25]. Development of DFU is related to chronic hyperglycemia, frequently in concert with other risk factors, for example, hypertension, low physical activity, and overweight/obesity [3]. Such factors contribute to development of peripheral neuropathy and peripheral artery disease which are typically the precursors to DFU [3]. Therefore, it is unsurprising that the scope of education reported to be provided by podiatrists encompassed content beyond direct footcare including diabetes and lifestyle management.

The findings of this review highlight limited available data investigating provision of diabetes footcare education by podiatrists in Australia and NZ and the limitations of survey methodologies. For better understanding of the current practices in provision of diabetes footcare education qualitative research design is required to describe the experiences, attitudes, and behaviors that drive both provision of education by podiatrists and how this is received by people living with diabetes [24]. These data will be key to determining health practitioner education requirements in relation to patient education and the comparative effectiveness of diabetes footcare education content and methods of education delivery.

## 5 LIMITATIONS

Robust search methods were used to conduct this review, however researchers in the field were not contacted for unpublished studies. It was planned that authors would only be contacted where it was considered that relevant data may have been collected as part of the study. In addition, gray literature was not searched, and this may have provided additional data in relation to provision of culturally responsive diabetes footcare education. Therefore, although this was not the primary aim of this review, it should be noted that this restricts the conclusions that can be drawn from the lack of data retrieved in relation to provision of culturally responsive diabetes footcare education. The lack of meta‐analysis of data relating to footcare education provision to the general community also limits the extent to which the study findings can be collectively interpreted.

## 6 CONCLUSION

Limited existing research shows that a broad range of education content is delivered by Australian and NZ podiatrists, who report providing footcare, specific and general diabetes education and covering multiple education topics in one consultation. Verbal education is used by the largest proportion of podiatrists in both countries and there is no significant difference between content mode of delivery and frequency of diabetes footcare education between private and public practitioners. Interpretation of existing data is limited to web‐based surveys, and a small pilot observational study, that represents a small proportion of registered practitioners in both countries. No studies have reported on the provision of culturally responsive diabetes footcare education provided by podiatrists in either country. Further research is required to establish the nature and delivery methods of diabetes footcare education, the alignment of diabetes related footcare education with current guidelines and the provision of culturally responsive diabetes footcare education and the availability of culturally responsive diabetes footcare education resources.

## AUTHOR CONTRIBUTIONS

**Maasooma Al Husaini**: Conceptualization; data curation; formal analysis; writing—original draft. **Angela Searle**: Data curation; writing—review and editing. **Vivienne Chuter**: Conceptualization; data curation; formal analysis; writing‐original draft; writing—review and editing; supervision.

## CONFLICT OF INTEREST STATEMENT

The authors of this review declare that there are no competing interests.

## ETHICS STATEMENT

Not applicable.

## Supporting information

Supporting Information S1

## Data Availability

Data supporting this study are included within the article and supporting information.
