# Supplementary material for: The composition and mode of delivery of diabetes‐related footcare education provided by podiatrists in Australia and Aotearoa (New Zealand): A systematic review
Source: J Foot Ankle Res. 2024 Nov 11;17(4):e70009. doi: 10.1002/jfa2.70009 (PMC11554429; doi:10.1002/jfa2.70009)
Supplement: Supplementary file 1 — Supporting Information S1 [file JFA2-17-e70009-s001.docx]

**Supplementary File 1**

**Search Strings**

Medline/Embase

1. exp Foot Ulcer/

2. exp Diabetic Foot/

3. (diabet* adj3 ulcer*).ti,ab.

4. (diabet* adj3 (foot or feet)).ti,ab.

5. 1 or 2 or 3 or 4

6. (education adj3 (diabet* or patient* or structured)).ti,ab.

7. (information adj3 (written or printed or oral or patient)).ti,ab.

8. (diabetes adj3 (footcare or foot-care or selfcare or self-care)).ti,ab.

9. (clinical adj3 practice).ti,ab.

10. (foot adj3 management).ti,ab.

11. 6 or 7 or 8 or 9 or 10

12. podiatr*.ti,ab.

13. 5 and 11 and 12

CINAHL

MH diabetic foot OR MH foot ulcer OR TI (diabet* N3 ulcer*) OR AB (diabet* N3 ulcer*) OR TI ( diabet* N3 (foot or feet) ) OR AB ( diabet* N3 (foot or feet) )

AND

( TI (education N3 (diabet* or patient* or structured)) ) OR ( AB (education N3 (diabet* or patient* or structured)) ) OR ( TI (information N3 (written or printed or oral or patient)) ) OR ( AB (information N3 (written or printed or oral or patient)) ) OR ( TI (diabetes N3 (footcare or foot-care or selfcare or self-care)) ) OR ( AB (diabetes N3 (footcare or foot-care or selfcare or self-care)) ) OR TI (clinical N3 practice) OR AB (clinical N3 practice) OR TI (foot N3 management) OR AB (foot N3 management)

AND

TI podiatr* AND AB podiatr*
